# Supplementary material for: Seroprevalence and silent infection rate during SARS-CoV-2 pandemic among children and adolescents in Western Pomerania: a multicenter, cross-sectional study—the COVIDKID study
Source: PeerJ. 2024 Nov 11;12:e18384. doi: 10.7717/peerj.18384 (PMC11562825; doi:10.7717/peerj.18384)
Supplement: Supplemental Information 1 [file peerj-12-18384-s001.docx]

**Sample size calculation**

A sample size calculation was performed and was part of the evaluation by the Ethics Committee in April 2020. We calculated that the number of children to be included should be 1,000 in order to estimate the silent infection rate with a precision of ± 2%. As the analysis was originally intended to show a semi-annual rate, it was planned to ask 6,000 children and adolescents for their participation in order to include at least 4,000 subjects. Given the dynamics of the pandemic, the anticipated number was not reached and the statistical analysis had to be modified to take sufficient account of variants, vaccinations and other risk factors.

**Evaluation of infection dynamics in the study region**

The analysis of the study includes the use of external data. In order to correlate the seroprevalence with the incidence of infection in the corresponding districts, we were provided in personal communication with data from the LaGuS (Landamt für Gesundheit und Soziales Mecklenburg-Vorpommern, https://www.lagus.mv-regierung.de/), which indicates the reported cases at district level for each individual year of life to allow the computation of age-dependent incidence rates. Centered 7-day averages of daily case numbers were used to compute the incidence of daily infections per 1,000 children in the respective age group. For this we assessed the number of children as of 31st December 2021 from official register data (LaiV: Landesamt für innere Verwaltung, Statistisches Amt, Table “A133K 2021 00”, https://d-nb.info/1261800559/34). Dates of key events and lockdown periods were obtained from the statistical year book 2022 (StatA MV, Statistisches Jahrbuch 2022, online publication Z011 2022 00, https://d-nb.info/022649670). Vaccination approval dates were obtained from European Medicines Agency (EMA).

Weekly reports of CoMV-Gen (https://www.comv-gen.de/) were used to assess the distribution of virus variants in order to set reasonable thresholds for the chronological division according to waves and variants. Here the change of the dominating SARS-CoV-2 variant in the federal state of MV was registered by V-PCR for the change from Alpha to Delta variant and whole genome sequencing for the change from dominant Delta to the Omicron variant. In personal communication, CoMV-Gen also provided detailed data on infections and variants in children and adolescents from SORMAS (https://sormas.org) to assess the dates of firstly detected variants in MV in these particular age groups. Turning dates of the new dominating variants were interpolated from weekly fractions of challenging variants (interpolated fraction >50% for respective variant). Accordingly, Delta-domination started from 20 June 2021 and Omicron-domination started from 06 January 2022 in the study region.

In order to check the representability of age and sex of our patients for all children across the three districts, we used official register data from LaiV: Landesamt für innere Verwaltung, Statistisches Amt (Table “A133K 2021 00”, https://d-nb.info/1261800559/34).
Furthermore, we checked whether the vaccination rate estimated from our study sample follows the pediatric vaccination rate in the three districts as tracked by the vaccination monitoring of the Robert-Koch-Institute which is a federal institute within the portfolio of the German Federal Ministry of Health (Robert Koch-Institut (2024): COVID-19-Impfungen in Deutschland, Berlin:Zenodo. DOI: 10.5281/zenodo.12581481, accessed 1 August 2024).

Zip codes of the residence address were assessed by questionnaire, aggregated across all patients and mapped to spatial locations of the respective zip code area. Spatial coordinates were gathered using Zauberware (https://github.com/zauberware/postal-codes-json-xml-csv, accessed 21 October 2022) that utilizes public data from Geonames (http://www.geonames.org/). Spatial polygons of GADM version 4.1 (https://gadm.org/data.html, accessed 6 August 2024) was further used to create maps of the origin of study participants.
