# Supplementary material for: Seroprevalence and silent infection rate during SARS-CoV-2 pandemic among children and adolescents in Western Pomerania: a multicenter, cross-sectional study—the COVIDKID study
Source: PeerJ. 2024 Nov 11;12:e18384. doi: 10.7717/peerj.18384 (PMC11562825; doi:10.7717/peerj.18384)
Supplement: Supplemental Information 2 [file peerj-12-18384-s002.docx]

**Table S1** Seroprevalences of the study population stratified by vaccination and infection status, COVIDKID December 2020 - August 2022

|  | Included children in Western Pomerania, Germany | | | | | | |
| --- | --- | --- | --- | --- | --- | --- | --- |
|  | **Vaccinated** (with and without infection)  *N=134* | | **Only diagnosed**  *N=162* | | **Unvaccinated and undiagnosed**  *N=806* | | |
| Variable | *N* | *Seroprevalence* | *N* | *Seroprevalence* | *N* | *Seroprevalence* | *P-value*^a^ |
| Age group, n/N (%) | 134 |  | 162 |  | 806 |  | 0.066 |
| Children under 5  (<5 years) |  | 1/2 (50.0%) |  | 33/44 (75.0%) |  | 18/220 (8.2%) |  |
| School children  (5-11 years) |  | 21/21 (100.0%) |  | 48/77 (62.3%) |  | 12/295 (4.1%) |  |
| Adolescents  (12-17 years) |  | 104/111 (93.7%) |  | 29/41 (70.7%) |  | 11/291 (3.8%) |  |
| Sex, n/N (%) | 134 |  | 162 |  | 806 |  | 0.75 |
| Male |  | 51/57 (89.5%) |  | 57/82 (69.5%) |  | 22/411 (5.4%) |  |
| Female |  | 75/77 (97.4%) |  | 53/80 (66.2%) |  | 19/395 (4.8%) |  |
| Sampling at, n/N (%) | 134 |  | 162 |  | 806 |  | <0.001 |
| Alpha waves  (10/Dec/20-19/Jun/21) |  | 2/3 (66.7%) |  | 6/11 (54.5%) |  | 9/446 (2.0%) |  |
| Delta waves  (20/Jun/21-05/Jan/22) |  | 24/30 (80.0%) |  | 17/20 (85.0%) |  | 13/244 (5.3%) |  |
| Omicron waves  (06/Jan/22-31/Aug/22) |  | 100/101 (99.0%) |  | 87/131 (66.4%) |  | 19/116 (16.4%) |  |
| Day care facility, n/N (%) | 119 |  | 152 |  | 757 |  | 0.25 |
| Not in a day care facility |  | 8/9 (88.9%) |  | 5/8 (62.5%) |  | 0/65 (0.0%) |  |
| Nursery/Nanny |  | 0/0 (NaN%) |  | 15/18 (83.3%) |  | 5/97 (5.2%) |  |
| Kindergarten |  | 4/5 (80.0%) |  | 25/34 (73.5%) |  | 9/130 (6.9%) |  |
| School with after-school care |  | 4/4 (100.0%) |  | 9/15 (60.0%) |  | 2/56 (3.6%) |  |
| School without after-school care |  | 96/101 (95.0%) |  | 47/77 (61.0%) |  | 19/409 (4.6%) |  |
| Medication intake at sampling, n/N (%) | 133 |  | 153 |  | 783 |  | 0.24 |
| No |  | 57/61 (93.4%) |  | 52/68 (76.5%) |  | 14/381 (3.7%) |  |
| Yes |  | 68/72 (94.4%) |  | 50/85 (58.8%) |  | 23/402 (5.7%) |  |
| Chronic diseases, n/N (%) | 131 |  | 154 |  | 782 |  | 0.048 |
| No chronic diseases |  | 76/82 (92.7%) |  | 74/114 (64.9%) |  | 26/568 (4.6%) |  |
| Chronic diseases except respiratory |  | 36/37 (97.3%) |  | 20/31 (64.5%) |  | 5/163 (3.1%) |  |
| Chronic diseases including respiratory |  | 11/12 (91.7%) |  | 8/9 (88.9%) |  | 6/51 (11.8%) |  |
| Smoking parent, n/N (%) | 131 |  | 157 |  | 791 |  | 0.18 |
| No |  | 80/84 (95.2%) |  | 69/102 (67.6%) |  | 24/417 (5.8%) |  |
| Yes |  | 43/47 (91.5%) |  | 36/55 (65.5%) |  | 13/374 (3.5%) |  |
| Child is smoking, n/N (%) | 132 |  | 157 |  | 795 |  | 0.40 |
| No |  | 110/115 (95.7%) |  | 101/150 (67.3%) |  | 37/760 (4.9%) |  |
| Yes/maybe |  | 14/17 (82.4%) |  | 4/7 (57.1%) |  | 0/35 (0.0%) |  |
| Household member works in the healthcare system with patient contact (including nursing home, nursing service and others), n/N (%) | 126 |  | 156 |  | 782 |  | 1.0 |
| No |  | 36/37 (97.3%) |  | 33/57 (57.9%) |  | 11/242 (4.5%) |  |
| Yes |  | 83/89 (93.3%) |  | 71/99 (71.7%) |  | 26/540 (4.8%) |  |
| Highest level of education of the household, n/N (%) | 122 |  | 151 |  | 579 |  | 0.69 |
| no high school diploma (yet) („(noch) kein Abschluss“) |  | 13/13 (100.0%) |  | 2/2 (100.0%) |  | 2/28 (7.1%) |  |
| Secondary school („Hauptschule“) |  | 7/8 (87.5%) |  | 8/9 (88.9%) |  | 2/38 (5.3%) |  |
| Junior high school (“Mittlere Reife”) |  | 36/38 (94.7%) |  | 36/57 (63.2%) |  | 15/250 (6.0%) |  |
| University entrance qualification („Hochschulreife“) |  | 26/27 (96.3%) |  | 31/40 (77.5%) |  | 5/120 (4.2%) |  |
| Academic degree („Hochschulabschluss“) |  | 33/36 (91.7%) |  | 25/43 (58.1%) |  | 12/143 (8.4%) |  |
| Number of household members, n/N (%) | 122 |  | 156 |  | 771 |  | 0.29 |
| 1 or 2 |  | 13/14 (92.9%) |  | 5/10 (50.0%) |  | 2/51 (3.9%) |  |
| 3 |  | 27/29 (93.1%) |  | 20/31 (64.5%) |  | 12/227 (5.3%) |  |
| 4 |  | 43/46 (93.5%) |  | 46/68 (67.6%) |  | 11/313 (3.5%) |  |
| 5 |  | 20/20 (100.0%) |  | 23/34 (67.6%) |  | 4/106 (3.8%) |  |
| 6 or more |  | 12/13 (92.3%) |  | 10/13 (76.9%) |  | 7/74 (9.5%) |  |
| A small child (<5 years) belongs to the household or is one of the very close contacts of the household community, n/N (%) | 68 |  | 83 |  | 586 |  | 0.27 |
| No |  | 57/61 (93.4%) |  | 44/64 (68.8%) |  | 16/444 (3.6%) |  |
| Yes |  | 6/7 (85.7%) |  | 11/19 (57.9%) |  | 2/142 (1.4%) |  |
| A senior (≥60 years) belongs to the household or is one of the very close contacts of the household community, n/N (%) | 68 |  | 83 |  | 586 |  | 0.23 |
| No |  | 31/33 (93.9%) |  | 26/41 (63.4%) |  | 8/342 (2.3%) |  |
| Yes |  | 32/35 (91.4%) |  | 29/42 (69.0%) |  | 10/244 (4.1%) |  |
| Animals/pets in the household, n/N (%) | 112 |  | 128 |  | 774 |  | 0.29 |
| No |  | 44/47 (93.6%) |  | 33/52 (63.5%) |  | 11/327 (3.4%) |  |
| Yes |  | 61/65 (93.8%) |  | 51/76 (67.1%) |  | 23/447 (5.1%) |  |
| Travelled beyond the district during the past 6 months, n/N (%) | 131 |  | 157 |  | 796 |  | 0.12 |
| No |  | 46/49 (93.9%) |  | 48/75 (64.0%) |  | 18/484 (3.7%) |  |
| Yes |  | 78/82 (95.1%) |  | 57/82 (69.5%) |  | 19/312 (6.1%) |  |
| Burdens of the respondent parent considered to be the most serious |  |  |  |  |  |  |  |
| Job insecurity, n/N (%) | 129 |  | 154 |  | 594 |  | 0.17 |
| No |  | 106/113 (93.8%) |  | 85/125 (68.0%) |  | 32/456 (7.0%) |  |
| Yes |  | 16/16 (100.0%) |  | 20/29 (69.0%) |  | 5/138 (3.6%) |  |
| Fear of their own illness, n/N (%) | 129 |  | 154 |  | 594 |  | 0.57 |
| No |  | 92/98 (93.9%) |  | 86/126 (68.3%) |  | 29/433 (6.7%) |  |
| Yes |  | 30/31 (96.8%) |  | 19/28 (67.9%) |  | 8/161 (5.0%) |  |
| Fear of illness in close relatives, n/N (%) | 129 |  | 154 |  | 594 |  | 0.39 |
| No |  | 61/63 (96.8%) |  | 62/90 (68.9%) |  | 24/338 (7.1%) |  |
| Yes |  | 61/66 (92.4%) |  | 43/64 (67.2%) |  | 13/256 (5.1%) |  |

^a^ Fisher‘s exact test for testing counts of unvaccinated and undiagnosed children
