# Supplementary figures and images for: Seroprevalence and silent infection rate during SARS-CoV-2 pandemic among children and adolescents in Western Pomerania: a multicenter, cross-sectional study—the COVIDKID study
Source: PeerJ. 2024 Nov 11;12:e18384. doi: 10.7717/peerj.18384 (PMC11562825; doi:10.7717/peerj.18384)

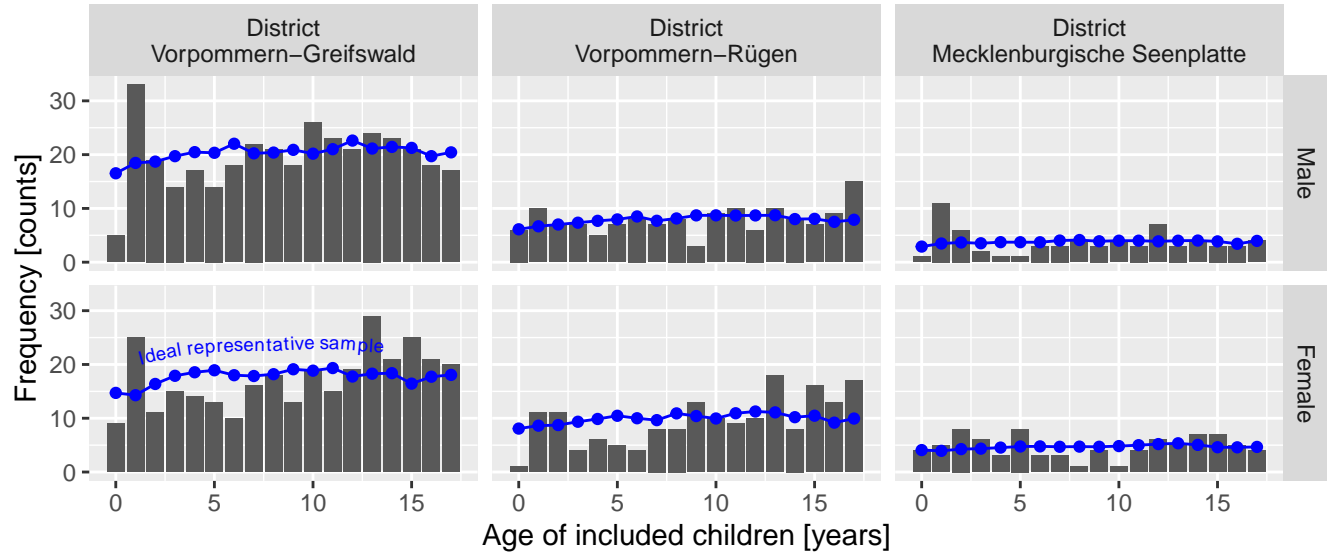

Supplement: Supplemental Information 3 — The blue line represents the sex-specific age distribution scaled to the total number of participants in the respective district, as reported by the Statistical Office Mecklenburg-Western Pomerania at the end of year 2021. [file peerj-12-18384-s003.pdf]

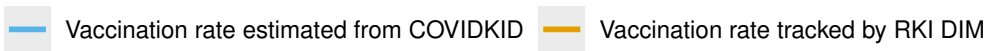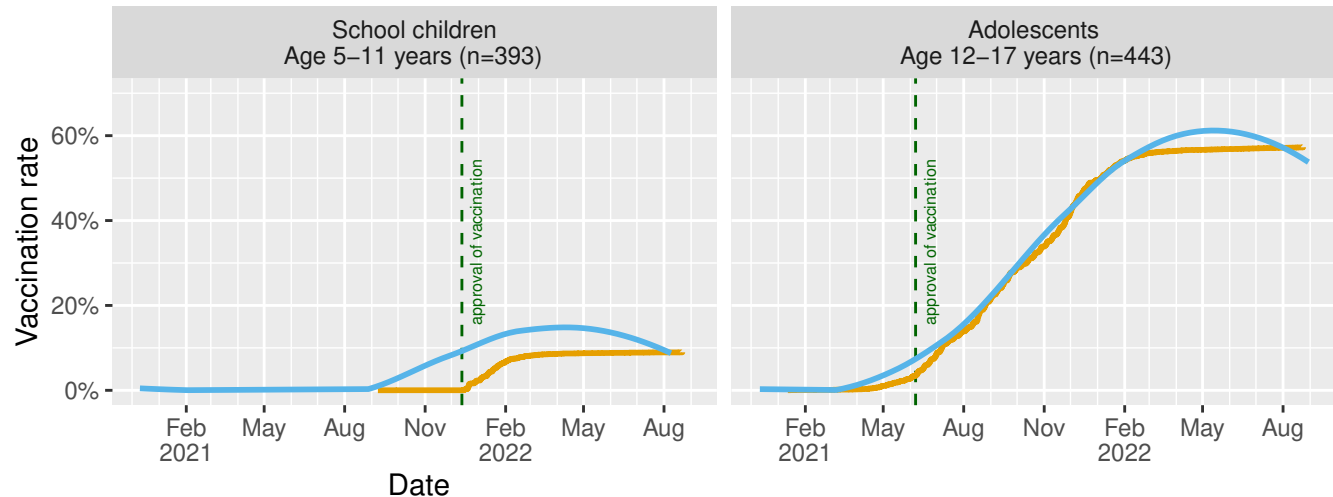

Supplement: Supplemental Information 4 — The COVIDKID vaccination rate estimation using the LOESS algortihm shows a similar curve to the vaccination rates in the respective age group of the three study districts as monitored by the Robert Koch Institute (RKI DIM). [file peerj-12-18384-s004.pdf]

# Origin of patients by zip code

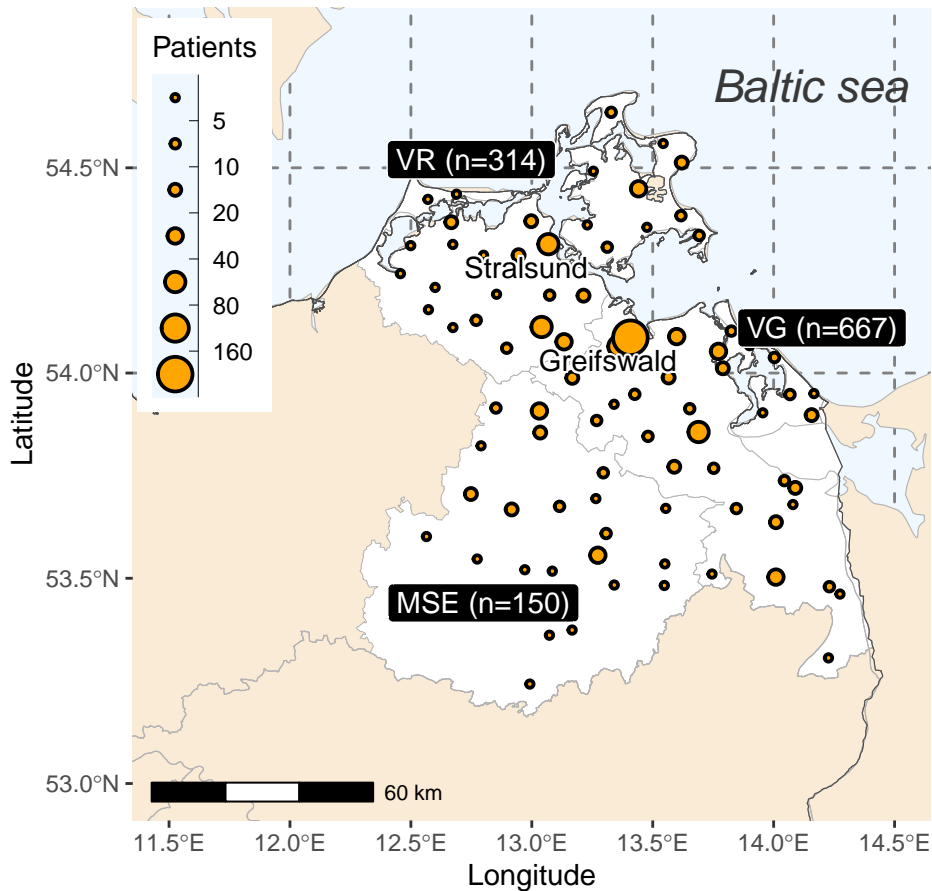

Supplement: Supplemental Information 5 — Number of participants by ZIP codes in the three districts of north-east German region Western Pomerania, COVIDKID December 2020 - August 2022; abbreviations: VR Vorpommern-Rügen, VG Vorpommern-Greifswald, MSE Mecklenburgische Seenplatte. [file peerj-12-18384-s005.pdf]

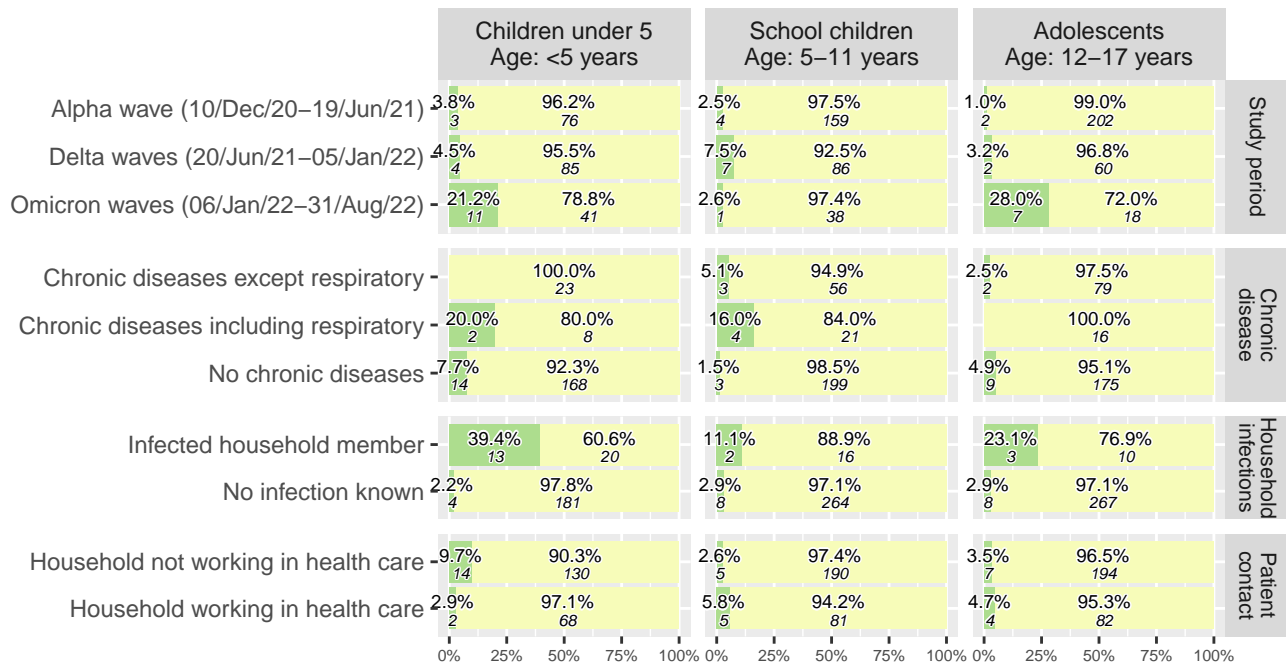

Supplement: Supplemental Information 7 — Seroprevalence stratified by age and depending on the time period, presence of chronic respiratory diseases, known household infections and possible patient contact. Green bars represent evident silent infections. [file peerj-12-18384-s007.pdf]
