# Supplementary material for: Seroprevalence and silent infection rate during SARS-CoV-2 pandemic among children and adolescents in Western Pomerania: a multicenter, cross-sectional study—the COVIDKID study
Source: PeerJ. 2024 Nov 11;12:e18384. doi: 10.7717/peerj.18384 (PMC11562825; doi:10.7717/peerj.18384)
Supplement: Supplemental Information 6 — The composition of seropositive samples is presented depending on self-disclosed infection and vaccination. The timeline below shows reported cases to the health department among children in the analyzed districts. Dashed lines show the official start of vaccination in the study region depending on the respective age group. Red areas show different levels of school lockdowns at the beginning of the study. [file peerj-12-18384-s006.pdf]

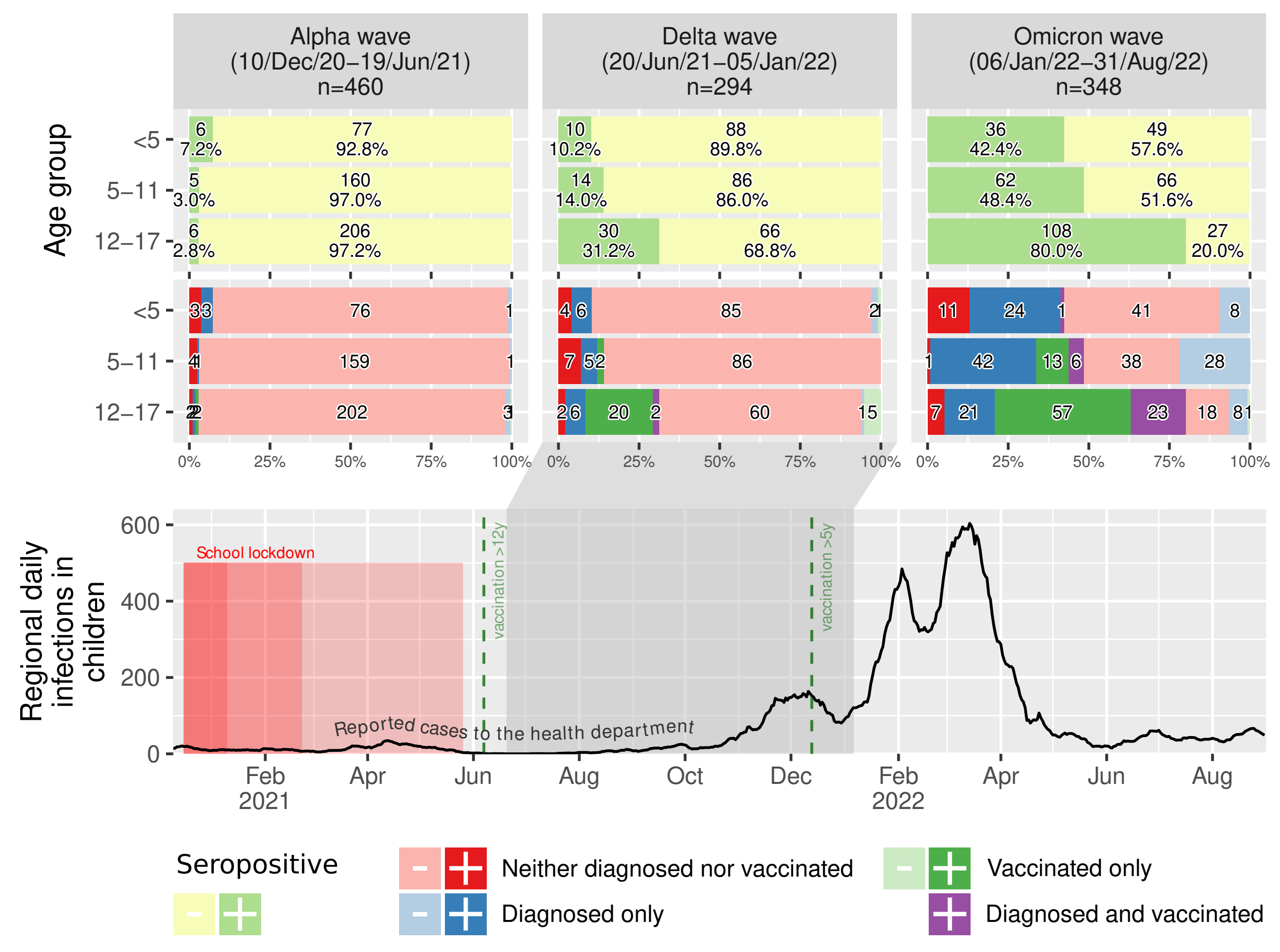

Alpha wave
(10/Dec/20–19/Jun/21)
n=460
Delta wave
(20/Jun/21–05/Jan/22)
n=294
Omicron wave
(06/Jan/22–31/Aug/22)
n=348
Age group
<5
5–11
12–17
0%
25%
50%
75%
100%
Regional daily infections in children
School lockdown
Reported cases to the health department
vaccination >12y
vaccination >5y
Feb 2021
Apr
Jun
Aug
Oct
Dec
Feb 2022
Seropositive
Neither diagnosed nor vaccinated
Diagnosed only
Vaccinated only
Diagnosed and vaccinated
