# Supplementary material for: Seroprevalence and silent infection rate during SARS-CoV-2 pandemic among children and adolescents in Western Pomerania: a multicenter, cross-sectional study—the COVIDKID study
Source: PeerJ. 2024 Nov 11;12:e18384. doi: 10.7717/peerj.18384 (PMC11562825; doi:10.7717/peerj.18384)
Supplement: Supplemental Information 9 [file peerj-12-18384-s009.docx]

STROBE Statement—checklist of items that should be included in reports of observational studies

|  | Item No. | Recommendation | Page  No. | Relevant text from manuscript |
| --- | --- | --- | --- | --- |
| **Title and abstract** | 1 | (*a*) Indicate the study’s design with a commonly used term in the title or the abstract | 1, 2 | multicenter, cross-sectional study |
|  |  | (*b*) Provide in the abstract an informative and balanced summary of what was done and what was found | 2, 3 | SI rate was 5.4% (95%-CI 3.7% to 6.8%) among unvaccinated and undiagnosed children... |
| Introduction | | | |  |
| Background/rationale | 2 | Explain the scientific background and rationale for the investigation being reported | 3 | In contrast to adults, children and adolescents are often only mildly affected by acute SARS-CoV-2 infection, with only mild or no symptoms, resulting in significant under-ascertainment rate. |
| Objectives | 3 | State specific objectives, including any prespecified hypotheses | 4 | ... aimed to analyze temporal changes of seroprevalence for SARS-CoV-2 ... |
| Methods | | | |  |
| Study design | 4 | Present key elements of study design early in the paper | 4 | For this multicenter, cross-sectional study, ... |
| Setting | 5 | Describe the setting, locations, and relevant dates, including periods of recruitment, exposure, follow-up, and data collection | 1, 4, 5, 6 | ... from December 2020 to August 2022.  SARS-CoV-2 specific antibodies in serum or plasma were analyzed ... |
| Participants | 6 | (*a*) *Cohort study*—Give the eligibility criteria, and the sources and methods of selection of participants. Describe methods of follow-up  *Case-control study*—Give the eligibility criteria, and the sources and methods of case ascertainment and control selection. Give the rationale for the choice of cases and controls  *Cross-sectional study*—Give the eligibility criteria, and the sources and methods of selection of participants | 4 | ... recruited in six participating pediatric hospitals and two outpatient practices ... during routine blood draw .... |
|  |  | (*b*) *Cohort study*—For matched studies, give matching criteria and number of exposed and unexposed  *Case-control study*—For matched studies, give matching criteria and the number of controls per case |  |  |
| Variables | 7 | Clearly define all outcomes, exposures, predictors, potential confounders, and effect modifiers. Give diagnostic criteria, if applicable | 5, 6 | SARS-CoV-2 specific antibodies in serum... |
| Data sources/ measurement | 8* | For each variable of interest, give sources of data and details of methods of assessment (measurement). Describe comparability of assessment methods if there is more than one group | 5, 6, Suppl. methods | Seropositivity was asserted when one of the following conditions was true: 1) positive IgG-S1; 2) ... |
| Bias | 9 | Describe any efforts to address potential sources of bias | 5, 6 | Official register data was used to check the representativeness of age, sex, and vaccination rate of our children’s sample.  To evaluate associating factors, multivariable logistic regressions were performed for OI and SI ...  We applied 4-fold cross-validated elastic net with a fair mix of L1- and L2-regularization (alpha=0.5) onto the imputed data frames to reduce model size for better generalizability...  Multicollinearity was checked... |
| Study size | 10 | Explain how the study size was arrived at | 4 | ... recruited in six participating pediatric hospitals and two outpatient practices ... during routine blood draw .... |

Continued on next page

| Quantitative variables | 11 | Explain how quantitative variables were handled in the analyses. If applicable, describe which groupings were chosen and why |  |  |
| --- | --- | --- | --- | --- |
| Statistical methods | 12 | (*a*) Describe all statistical methods, including those used to control for confounding | 6 | Three study periods were defined based on dominating variant...  The cumulative number of infections was normalized by the number of children in each age group of the three districts to adjust for the risk of being infected so far. |
|  |  | (*b*) Describe any methods used to examine subgroups and interactions | 6 | ...multivariable logistic regression were performed for OI and SI...  We performed Type-III likelihood ratio tests and computed odds ratios ... |
|  |  | (*c*) Explain how missing data were addressed | 6 | Since this requires complete data, we created 10 imputed data frames using random forest imputation.  ...all vaccinated participants and those with incomplete information about their vaccination or infection history were excluded. |
|  |  | (*d*) *Cohort study*—If applicable, explain how loss to follow-up was addressed  *Case-control study*—If applicable, explain how matching of cases and controls was addressed  *Cross-sectional study*—If applicable, describe analytical methods taking account of sampling strategy | N/A |  |
|  |  | (*e*) Describe any sensitivity analyses | N/A |  |
| Results | | | | |
| Participants | 13* | (a) Report numbers of individuals at each stage of study—eg numbers potentially eligible, examined for eligibility, confirmed eligible, included in the study, completing follow-up, and analysed | 4, 7 | Three patients with known immunodeficiencies or administration of immunoglobulins were excluded.  In total, 1,166 samples from 1,093 children and adolescents were included. |
|  |  | (b) Give reasons for non-participation at each stage | N/A |  |
|  |  | (c) Consider use of a flow diagram | N/A |  |
| Descriptive data | 14* | (a) Give characteristics of study participants (eg demographic, clinical, social) and information on exposures and potential confounders | 7, 8 | In total, 1,131 samples ...  Participants characteristics and serological test results stratified by age are summarized in Table 1. |
|  |  | (b) Indicate number of participants with missing data for each variable of interest | Table 1 | Missing values are included in the Tables |
|  |  | (c) *Cohort study*—Summarise follow-up time (eg, average and total amount) | NA |  |
| Outcome data | 15* | *Cohort study*—Report numbers of outcome events or summary measures over time | NA |  |
|  |  | *Case-control study—*Report numbers in each exposure category, or summary measures of exposure | NA |  |
|  |  | *Cross-sectional study—*Report numbers of outcome events or summary measures | 8, 9 | In the group of SARS-CoV-2 undiagnosed and unvaccinated participants, 4.1% were positive for IgG-S1 (33/ 806).  IIn the presumptive negative samples, 41 undetected infections were confirmed, which corresponds to an SI rate of 5.4% (41/806, 95% Clopper-Pearson confidence interval 3.7% to 6.8%, see Fig. 2b). |
| Main results | 16 | (*a*) Give unadjusted estimates and, if applicable, confounder-adjusted estimates and their precision (eg, 95% confidence interval). Make clear which confounders were adjusted for and why they were included | Table3, 10, 11 | OI was significantly associated with infection risk ... |
|  |  | (*b*) Report category boundaries when continuous variables were categorized | 5 | Samples were considered positive at a ratio ≥1.1, as recommended by the manufacturer.  ... inhibition with ≥30% inhibition capacity classified as positive. |
|  |  | (*c*) If relevant, consider translating estimates of relative risk into absolute risk for a meaningful time period | NA |  |

Continued on next page

| Other analyses | 17 | Report other analyses done—eg analyses of subgroups and interactions, and sensitivity analyses | 5, 7-11 | Official register data was used...  Turning dates of the new dominating variants were interpolated... |
| --- | --- | --- | --- | --- |
| Discussion | | | | |
| Key results | 18 | Summarise key results with reference to study objectives | 11, 16 | In our study we aimed to investigate ...  In the study sample, the rate of SI in undiagnosed and unvaccinated children and adolescents was 5.4%. |
| Limitations | 19 | Discuss limitations of the study, taking into account sources of potential bias or imprecision. Discuss both direction and magnitude of any potential bias | 15 | There are also some limitations of our study. ... |
| Interpretation | 20 | Give a cautious overall interpretation of results considering objectives, limitations, multiplicity of analyses, results from similar studies, and other relevant evidence | 13-16 | The revised test strategy in schools may have led to early and rapid detection of infections... |
| Generalisability | 21 | Discuss the generalisability (external validity) of the study results | 13-16 | This is in line with comparable studies across Europe...  Other seroprevalence studies from Germany showed similar results for children in different age groups, ... |
| Other information | |  | | |
| Funding | 22 | Give the source of funding and the role of the funders for the present study and, if applicable, for the original study on which the present article is based | 17 | This study was funded by...  The sponsor was not involved in ... |

*Give information separately for cases and controls in case-control studies and, if applicable, for exposed and unexposed groups in cohort and cross-sectional studies.

**Note:** An Explanation and Elaboration article discusses each checklist item and gives methodological background and published examples of transparent reporting. The STROBE checklist is best used in conjunction with this article (freely available on the Web sites of PLoS Medicine at http://www.plosmedicine.org/, Annals of Internal Medicine at http://www.annals.org/, and Epidemiology at http://www.epidem.com/). Information on the STROBE Initiative is available at www.strobe-statement.org.
